# Using protection motivation theory to predict monkeypox vaccination intention among adults in Chongqing, China

**DOI:** 10.3389/fimmu.2026.1871884

**Published:** 2026-08-17

**Authors:** Wenshuang Wei, Ju Wang, Rui Wu, Yu Xiong, Ke Zhang, Xiaoli Tang, Huadong Zhang, Jiang Long, Xiao Tang, Xihao Cheng, Li Qi

**Affiliations:** 1 Department of Infectious Disease Prevention and Control,Chongqing Municipal Center for Disease Control and Prevention, Chongqing, China; 2 Ministry of Basic Medical Education, Dazhou Vocational College of Chinese medicine, Dazhou, Sichuan, China; 3 Department of Infectious Disease Prevention and Control,Yuzhong District Center for Disease Control and Prevention, Chongqing, China; 4 Department of Infectious Disease Prevention and Control,Beibei District Center for Disease Control and Prevention, Chongqing, China; 5 Chongqing Municipal Academy of Preventive Medicine, Chongqing, China; 6 School of Wellness and Leisure Studies, Tourism College of Zhejiang, Hangzhou, Zhejiang, China

**Keywords:** cross-sectional study, monkeypox, protection motivation theory, structural equation modeling (SEM), vaccination intention

## Abstract

**Background:**

The global spread of monkeypox represents a significant public health challenge. Vaccination is the cornerstone of pandemic control, and understanding the psychological factors underlying vaccination decisions is crucial. This study aimed to investigate the predictors of monkeypox vaccination intention among Chinese adults.

**Methods:**

A cross-sectional online survey was conducted among a convenience sample of adults in Chongqing, China, from March to April 2025. The questionnaire assessed sociodemographic characteristics, knowledge of monkeypox, and constructs from the Protection Motivation Theory (PMT). Structural equation modeling (SEM) was employed to test the direct and indirect paths between the PMT constructs and vaccination intention.

**Results:**

Of 805 valid respondents, 290 intended to vaccinate. CFA confirmed good reliability/validity of PMT scales (Cronbach’s α: 0.789–0.937). SEM showed good fit (CFI = 0.97, RMSEA = 0.04). Severity perception (β=0.14) and self-efficacy (β=0.13) positively predicted intention, while response cost (β=-0.26) negatively predicted it (all p<0.05). Knowledge had indirect effects via risk perception, response efficacy, and self-efficacy. Age was directly negatively associated with the vaccination intention.

**Conclusion:**

During the study period, no monkeypox vaccine had been approved for marketing in China, and routine vaccination was not recommended for the general public; in this context, willingness to receive a monkeypox vaccine among adults in Chongqing was relatively low. The Protection Motivation Theory provides a valuable framework for understanding vaccination intention. If vaccines are included in future routine recommendations, interventions should emphasize severity, reduce barriers, and build public confidence to increase uptake.

## 1 Introduction

Monkeypox is an emerging zoonotic disease caused by the monkeypox virus, which belongs to the *orthopoxvirus* genus within the *Poxviridae* family (1). The monkeypox virus was first discovered in monkeys in 1958, and the first human infection was reported in the Democratic Republic of Congo in 1970 (2, 3). Subsequently, the disease transitioned to an endemic state in Central and West Africa (4). In 2022, the World Health Organization (WHO) declared the multi-country monkeypox outbreak a Public Health Emergency of International Concern (PHEIC). Over the course of the outbreak, more than 99,000 confirmed cases were reported across 122 countries, and at least 100 countries reported their first mpox cases (5). On 14 August 2024, the WHO Director-General determined that the upsurge of mpox in the Democratic Republic of the Congo and a growing number of countries in Africa constituted a PHEIC, marking the second mpox-related PHEIC in two years (6). The declaration highlights the severity and risk of international spread, particularly the risk of spread beyond historically endemic regions (5). During the first PHEIC, China documented only a single imported case of monkeypox in September 2022, before a steady increase in confirmed cases thereafter (7, 8). The rise of monkeypox has underscored concerns about public health preparedness in China (9).

Evidence indicates that prior smallpox vaccination can significantly lower the risk of severe monkeypox infection (10). Vaccination intention against monkeypox varies widely across regions (11), with reported rates of 8.8% (12), 29.3% (13), 67% (4) and 68.88% (14). Still, vaccination uptake has been a persistent difficulty for nearly all countries. Despite steadily increasing vaccination coverage due to government efforts, hesitancy persists among some individuals, for different reasons (e.g., knowledge about monkeypox, education, income and parents of color) (15, 16). Initial vaccination willingness is a primary driver of subsequent vaccine uptake (17). Weak vaccination intention and vaccine hesitancy are key barriers to vaccination programmes (18). Early experience with coronavirus disease-2019 (COVID-19) reveals vaccine hesitancy exacerbated the epidemic (19).

Protection Motivation Theory (PMT) is a theoretical framework in social psychology aimed at health behavioral intentions, explaining how individuals cope with and respond to perceived health threats (20). According to PMT, intention is the direct determinant of behavior, shaped by two parallel processes: threat appraisal and coping appraisal. Threat appraisal is influenced by one’s perception of the severity of the health threat, their susceptibility to it, and the perceived benefits of any maladaptive behaviors. Coping appraisal is determined by beliefs about the effectiveness of the recommended response (response efficacy), confidence in one’s ability to perform it (self-efficacy), and the perceived barriers (response cost). Protection motivation (i.e., intention) arises from these two appraisal processes and is seen to be the sole determinant of protective behaviour, providing a mediating pathway for the effects of other PMT variables and more distant influences (e.g., demographics) (18).

PMT has been applied to investigate the acceptance of vaccines against many infectious diseases, like influenza (21), COVID-19 (22), hepatitis B (23), and human papillomavirus (24). Evidence from these studies has shown that this model is a good predictor of vaccination intention. The Protection Motivation Theory has been used in existing studies to examine monkeypox vaccination hesitancy among men who have sex with men population (MSM) (25). Although current epidemiological data indicate a disproportionate burden of transmission among MSM, evidence from outbreaks in West and Central Africa demonstrates the virus’s established capacity for transmission within the general population (26).

Currently, there remains limited understanding of public awareness, preventive behaviors, and perceptions regarding monkeypox, along with insufficient data on vaccination willingness. This study therefore aimed to investigate the intention to receive monkeypox vaccination among the general population of a city in western China, as well as related knowledge and behavioral predictors. To our knowledge, no prior research has specifically explored factors influencing monkeypox vaccination intention using the Protection Motivation Theory (PMT) in a general Chinese population. Therefore, this study aimed to: (1) estimate the prevalence of monkeypox vaccination intention among adults in Chongqing, China; (2) test the direct effects of PMT constructs (threat and coping appraisals) on vaccination intention using structural equation modeling; and (3) quantify the indirect effects of sociodemographic characteristics through PMT mediators. Our findings are expected to provide evidence for the development of behavioral interventions.

## 2 Methods

### 2.1 Study design and participants

This cross-sectional study was conducted in Chongqing, China, between March and April 2025. A two-stage district selection was used to ensure geographic coverage, but the final participant recruitment was conducted via convenience online sampling. First, three districts were randomly selected from the nine core districts of Chongqing’s primary urban area. Subsequently, considering the feasibility of online recruitment, a convenience sample of participants was recruited from these districts. Data were collected through an online survey hosted on the *wjx.cn* platform. Electronic informed consent was obtained from all participants on the first page of the online survey before they could proceed to the questionnaire items.

Eligible participants were required to meet the following criteria: (i) aged 18 years or older; (ii) Chinese citizens residing in Chongqing for over six months; and (iii) able to complete the questionnaire independently. All data collected were fully anonymized to ensure participant confidentiality.

### 2.2 Sample size

The minimum sample size was calculated using the formula for cross-sectional studies:


$$n=\frac{{{\text{Z}}_{1-\alpha /2}}^{2}\times \text{P}\times (1-\text{P})}{d^{2}}$$

Where Z = 1.96 (corresponding to a 95% confidence level), P = 0.5 (to maximize the sample size and ensure a conservative estimate), and d = 0.05 (margin of error). This calculation yielded a minimum sample size of 384. To account for the cluster sampling design, the sample size was adjusted using a design effect (DEFF) of 2, resulting in a final target sample size of 768 participants.

### 2.3 Measures

The PMT items were adapted from previously validated instruments (25, 27–29), whereas the knowledge section was self-developed based on national guidelines. A small-scale pilot study (n = 73) was then conducted, yielding an overall Cronbach’s α of 0.931, indicating good internal consistency of the questionnaire. Details of the item sources, translation, and content-validity procedures are provided in Section 2.3.3. The final questionnaire comprised the following four sections:

#### 2.3.1 Demographics

The demographics included the following items: gender (male/female), age (collected as a continuous variable, in years), occupation as a healthcare worker (yes/no), education level (primary or below/secondary/college or above), marital status (married/others), household per capita monthly income in Chinese Yuan (CNY;<2,500/2,500–7,500/>7,500), presence of chronic diseases (yes/no), and health insurance status (uninsured/insured).

#### 2.3.2 Knowledge

The knowledge section was composed of ten items, in terms of etiology, transmission, susceptible population, clinical manifestations, and prevention and treatment of monkeypox. It was based on Guidelines for the Diagnosis and Treatment of Monkeypox (2022 edition). The knowledge assessment module employed a tripartite response format (True/False/Unknown). Responses were coded using binary scoring (1 = correct response, 0 = false/unknown response), with total scores ranging from 0 to 10. Following the approach used in previous surveys of monkeypox knowledge among the general population in China (15), a cutoff score of 6 (correct response rate ≥ 60%) was used to describe the distribution of knowledge levels within the sample (high-knowledge group vs. low-knowledge group). In all inferential analyses (Pearson correlation analysis, between-group comparisons, and structural equation modeling), knowledge was treated as a continuous variable (0–10 points).

#### 2.3.3 PMT

The PMT section assessed two appraisal pathways: threat appraisal and coping appraisal. The threat appraisal was composed of three subconstructs: risk perception (3 items), severity perception (4 items) and fear arousal (4 items). The coping appraisal was also composed of three subconstructs: response efficacy (4 items), response cost (4 items) and self-efficacy (5 items). Each item was assessed with a 5-point Likert scale, ranging from “1=strongly disagree” to “5=strongly agree”. Higher scores indicated higher levels of the corresponding construct.

The PMT items were adapted from previously validated instruments (25, 27–29), translated and back-translated (English–Chinese) by two independent bilingual researchers, and reviewed for content validity by three senior public health experts. The complete list of PMT items is provided in **Supplementary Table 2**.

Reliability and validity analyses were conducted for the PMT items. The KMO value was 0.902, the p-value for Bartlett’s test was less than 0.001 and the total variance of the dimensions was 72.03%. The Cronbach’s α coefficients of risk perception, severity perception, fear arousal, response efficacy, response cost and self-efficacy were 0.789, 0.902, 0.857, 0.880, 0.794 and 0.937, respectively; the overall Cronbach’s α coefficient was 0.920. These results showed good reliability and validity of the scale.

#### 2.3.4 Intention

Vaccination willingness was defined as the self-reported likelihood of vaccine acceptance, measured utilizing a 5-point Likert scale. The scale was anchored from 1 (completely unwilling) to 5 (fully willing), with ascending values reflecting greater propensity for vaccine acceptance. In descriptive analyses (such as reporting the proportion of participants willing to be vaccinated), participants who responded with a score of 4 (“willing”) or 5 (“fully willing”) were classified as “willing,” while those who responded with a score of 1, 2, or 3 were classified as “unwilling.” All inferential analyses (Pearson correlation analysis and structural equation modeling) used the raw 5-point Likert scores as continuous variables, and their approximate normal distribution was confirmed prior to analysis.

### 2.4 Statistical analyses

Statistical analyses were conducted using IBM SPSS software version 27 and AMOS software version 28. Continuous variables were presented as means ± standard deviations (SD), and categorical variables were expressed as frequencies and proportions. Student’s t-test or Chi-square test was employed to compare differences between respondents in the willing and unwilling groups. Structural equation modeling (SEM) was used to assess the fit between the gathered data and the proposed model. Six indices were used to evaluate if the proposed model was supported, including GFI (> 0.90), AGFI (> 0.90), CFI (> 0.90), IFI (> 0.90), RMSEA (< 0.08), and chi-square/df ratio (< 3.0). A two-sided p-value of< 0.05 was considered significant for all tests. To ensure consistency in the analysis, vaccination willingness was treated as a continuous variable (raw scores of 1-5) in all Pearson correlation analyses and structural equation models. The dichotomous “willing/unwilling” variable was used only for descriptive comparisons between groups (**Tables 1**, **2**).

**Table 1:** The demographic characteristics of the study population.

Variable	Vaccination intention^b^		Total, n (%)
	Unwilling	Willing	
Sample size, N (%)	515 (63.98)	290 (36.02)	805 (100)
Age in years, n (%)			
18-30	117 (22.72)	94 (32.41)	211 (26.21)
31-40	126 (24.47)	77 (26.55)	203 (25.22)
41-50	116 (22.52)	46 (15.86)	162 (20.12)
≥51	156 (30.29)	73 (25.17)	229 (28.45)
Mean (SD)	43.82 (14.81)	40.09 (14.27)	42.48 (14.72)
Gender, n (%)			
Male	256 (49.71)	128 (44.14)	384 (47.70)
Female	259 (50.29)	162 (55.86)	421 (52.30)
Healthcare workers, n (%)			
No	410 (79.61)	209 (72.07)	619 (76.89)
Yes	105 (20.39)	81 (27.93)	186 (23.11)
Education level, n (%)			
Primary or below	116 (22.52)	42 (14.48)	158 (19.63)
Secondary	87 (16.89)	46 (15.86)	133 (16.52)
College or above	312 (60.58)	202 (69.66)	514 (63.85)
Marital status, n (%)			
Married	371 (72.04)	198 (68.28)	569 (70.68)
Others^a^	144 (27.96)	92 (31.72)	236 (29.32)
Monthly income, CNY, n (%)			
<2500	236 (45.83)	109 (37.59)	345 (42.86)
2500-7500	185 (35.92)	129 (44.48)	314 (39.01)
>7500	94 (18.25)	52 (17.93)	146 (18.14)
Insurance status, n (%)			
No	7 (1.36)	4 (1.38)	11 (1.37)
Yes	508 (98.64)	286 (98.62)	794 (98.63)
Chronic diseases, n (%)			
No	394 (76.50)	229 (78.97)	623 (77.39)
Yes	121 (23.50)	61 (21.03)	182 (22.61)

^a^ Others include divorced, widowed, and unmarried.

^b^ Vaccination willingness is defined as follows: a Likert scale response of 4 or 5 (“willing” or “fully willing”) is classified as “willing,” while a response of 1–3 is classified as “unwilling.”

**Table 2:** Knowledge of monkeypox.

Total scale/single item (correct answer)	Vaccination intention^a^		P value^b^
	Unwilling	Willing	
Total scale score, mean (SD)	5.32 (2.90)	6.78 (2.44)	<0.001
K1 Monkeypox is an infectious disease, n (%)	413 (80.19)	259 (89.31)	<0.001
K2 Monkeypox is not a newly discovered disease in 2022, n (%)	78 (15.15)	57 (19.66)	0.100
K3 The population is generally susceptible to monkeypox, n (%)	254 (49.32)	209 (72.07)	<0.001
K4 People who have sex with men are more susceptible, n (%)	271 (52.62)	202 (69.66)	<0.001
K5 Washing hands and wearing a mask are effective means of preventing monkeypox, n (%)	338 (65.63)	250 (86.21)	<0.001
K6 Currently, there are specific drugs for the treatment of monkeypox in China, n (%)	106 (20.58)	77 (26.55)	0.052
K7 The monkeypox vaccine can be used to prevent monkeypox infection, n (%)	313 (60.78)	232 (80.00)	<0.001
K8 Sources of infection with monkeypox include rodents and humans infected with the monkeypox virus, n (%)	296 (57.48)	221 (76.21)	<0.001
K9 Monkeypox can be contracted by touching rashes, crusts, or body fluids from an infected person, n (%)	359 (69.71)	236 (81.38)	<0.001
K10 Monkeypox infection is associated with typical skin lesions, n (%)	312 (60.58)	222 (76.55)	<0.001

^a^ Vaccination willingness is defined as follows: a Likert scale response of 4 or 5 (“willing” or “fully willing”) is classified as “willing,” while a response of 1–3 is classified as “unwilling”.

^b^ The comparisons between groups were performed using an independent samples t-test for the total knowledge score and Chi-square tests for individual knowledge items.

Confirmatory factor analysis (CFA) was conducted separately prior to structural equation modeling to test the measurement model. Parameter estimates were obtained using maximum likelihood (ML) estimation. The model was not modified based on modification indices, and correlated residuals were not allowed. All PMT items used a 5-point Likert scale; indirect effects were estimated using the Bootstrap method (5,000 resamples), and bias-corrected 95% confidence intervals were reported.

## 3 Results

### 3.1 Demographic characteristics

A total of 822 questionnaires were received, of which 805 were fully completed and met the inclusion criteria. Among these 805 qualified responses, 290 (36.02%) indicated intention to be vaccinated. The average age of the study population was 42.48 (SD = 14.72). Among them, 421 (52.30%) were female and 384 (47.70%) were male. Nearly two-thirds (63.85%) of respondents had a college degree or higher. The majority (70.68%) of the respondents were married. Additionally, 186 (23.11%) respondents were healthcare workers. In addition, 22.61% of respondents had a chronic disease.

### 3.2 Confirmatory factor analysis

**Table 3** presents the results of the reliability and validity analyses of the measurement model. All constructs demonstrated good internal consistency, with Cronbach’s α ranging from 0.789 to 0.937. Together with the strong evidence of previously reported structural validity (KMO = 0.902; Total Variance Explained = 72.03%), these results confirm that the scale used in this study possesses good reliability and validity for measuring the intended constructs.

**Table 3:** Descriptive statistics, factor loadings and Cronbach’s α for the PMT scales.

Measures	Items	Item mean ± SD	Subscale total mean ± SD	Standardized factor loading	Cronbach’s α
Risk perception	RP1	2.309±0.965	7.805±2.493	0.911	0.789
	RP2	2.357±0.985		0.916	
	RP3	3.139±1.022		0.594	
Severity perception	SP1	3.431±1.028	14.329±3.670	0.789	0.902
	SP2	3.450±1.022		0.821	
	SP3	3.711±1.067		0.842	
	SP4	3.738±1.058		0.819	
Fear arousal	FA1	2.616±0.983	10.907±3.552	0.725	0.857
	FA2	2.948±1.043		0.713	
	FA3	2.552±1.070		0.847	
	FA4	2.791±1.144		0.779	
Response efficacy	RE1	3.047±0.948	13.157±3.189	0.567	0.880
	RE2	3.343±0.935		0.547	
	RE3	3.498±0.919		0.648	
	RE4	3.268±0.914		0.534	
Response cost	RC1	3.276±0.828	11.602±2.721	0.533	0.794
	RC2	2.819±0.888		0.791	
	RC3	2.785±0.886		0.815	
	RC4	2.723±0.858		0.768	
Self-efficacy	SE1	3.043±0.899	15.071±3.910	0.808	0.937
	SE2	3.071±0.875		0.865	
	SE3	3.000±0.889		0.873	
	SE4	2.975±0.849		0.828	
	SE5	2.981±0.859		0.865	

### 3.3 Knowledge about monkeypox

The mean total knowledge score was 5.84 (standard deviation 2.83; range 0–10). Using 6 as the cutoff value, 519 participants (64.47%) were classified into the high-knowledge group, and 286 (35.53%) were classified into the low-knowledge group. The total knowledge score (as a continuous variable) was significantly higher in the willing group than in the unwilling group (6.78 ± 2.44 vs. 5.32 ± 2.90, p< 0.001; **Table 2**), and this trend was reflected in the vast majority of specific knowledge items, such as understanding of transmission routes, preventive measures, and vaccine effectiveness (p< 0.001).

### 3.4 Correlations between vaccination intention and PMT constructs

A Pearson correlation analysis using the raw 5-point Likert scale scores for vaccination willingness (1–5 points) showed significant associations between vaccination intention and PMT variables (**Table 4**): risk perception (r=0.34, p< 0.01), severity perception (r=0.29, p< 0.01), fear arousal (r=0.11, p< 0.01), response efficacy (r=0.40, p<0.01), response cost (r =-0.03, p< 0.01), and self-efficacy (r=0.47, p<0.01).

**Table 4:** Correlation between Monkeypox vaccination intention, PMT subconstructs.

Variables	X1	X2	X3	X4	X5	X6	X7
X1 Risk perception	1.00	0.33^**^	0.24^**^	0.38^**^	0.20^**^	0.40^**^	0.34^**^
X2 Severity perception		1.00	0.33^**^	0.60^**^	0.27^**^	0.39^**^	0.29*^*^
X3 Fear arousal			1.00	0.44^**^	0.55^**^	0.32^**^	0.11^**^
X4 Response efficacy				1.00	0.34^**^	0.63^**^	0.40^**^
X5 Response cost					1.00	0.24^**^	-0.03^**^
X6 Self-efficacy						1.00	0.47^**^
X7 Intention							1.00

^**^ p < 0.01, ^*^p < 0.05.

### 3.5 Analysis of SEM

We further assessed the convergent and discriminant validity of the measurement model. Composite reliability (CR) and average variance extracted (AVE) were calculated based on the standardized factor loadings of each item in **Table 3**. With the exception of response efficacy, all constructs had CR values greater than 0.8 and AVE values greater than 0.5 (**Supplementary Table 1**). Discriminant validity was assessed using the Fornell-Larcker criterion, which requires that the square root of the AVE for each construct be greater than its correlation coefficient with other constructs. With the exception of response efficacy, all constructs met this criterion (**Supplementary Table 3**). The convergent validity (AVE = 0.33) and discriminant validity of response efficacy did not meet the recommended standards, suggesting limitations in the measurement of this construct.

**Figure 1:**
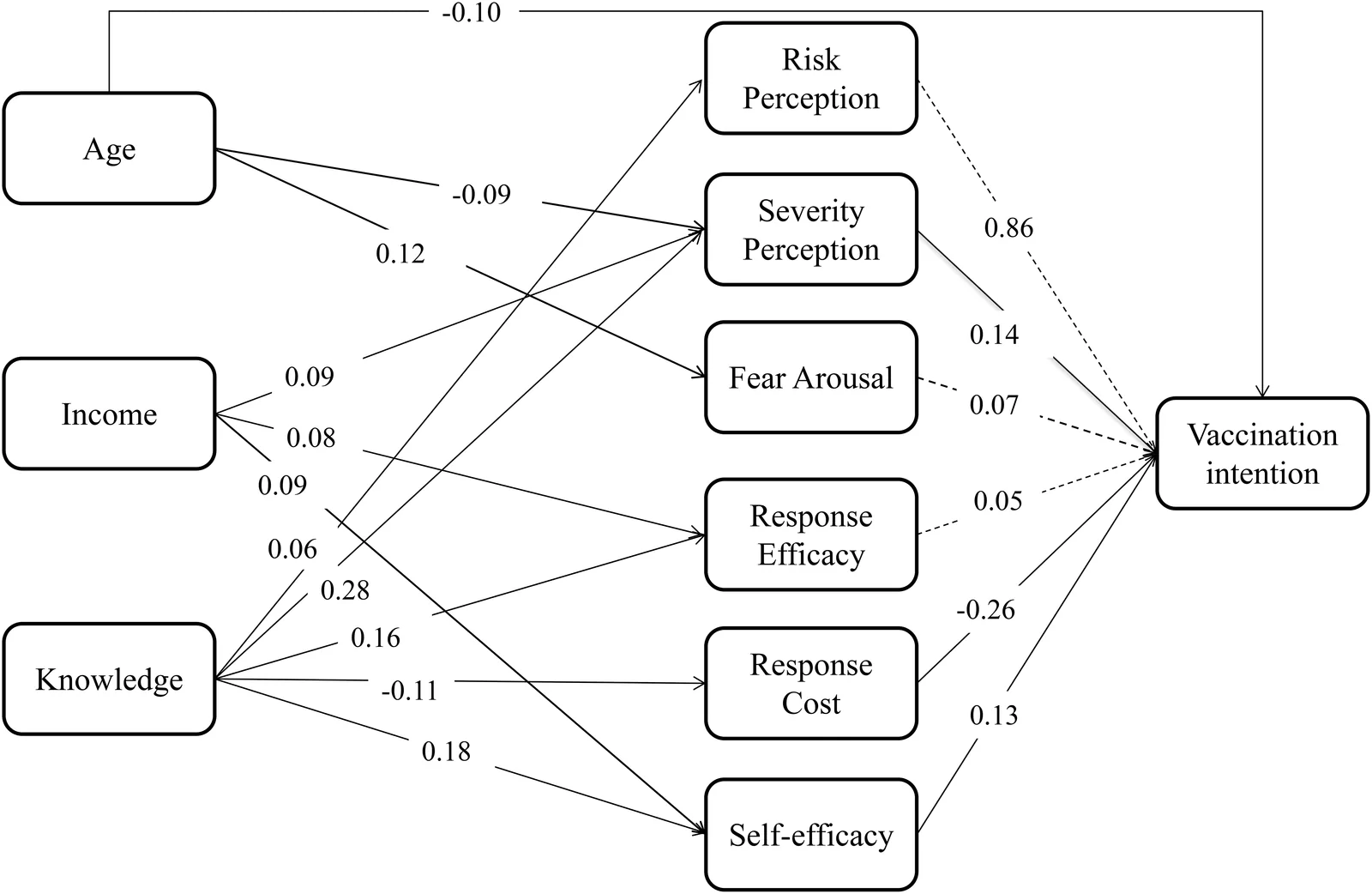
Path diagram of the model predicting vaccination intention.

**Figure 1** displays the structural equation modeling results. The model demonstrated an adequate fit to the data, as indicated by the following indices: GFI = 0.94, AGFI = 0.92, CFI = 0.97, IFI = 0.97, RMSEA = 0.04, and χ²/df = 2.46. Among the six PMT constructs, severity perception (β = 0.14, p< 0.05), response cost (β = -0.26, p< 0.05), and self-efficacy (β = 0.13, p< 0.05) exerted significant effects on monkeypox vaccination intention. Here, severity perception reflects the perceived seriousness of monkeypox consequences, response cost pertains to the perceived barriers to vaccination and self-efficacy denotes the confidence in one’s ability to receive vaccination. Age had a significant negative direct effect on vaccination intentions (β = -0.10, p< 0.05).

Analysis of indirect effects revealed that knowledge exerts a significant indirect effect on vaccination willingness through risk perception, response efficacy, and self-efficacy; income exerts a significant indirect effect on vaccination willingness through response efficacy and self-efficacy. Detailed results for all indirect effects are presented in **Supplementary Table 5**.

## 4 Discussion

This study employed the PMT to examine monkeypox vaccination intention among adults in Chongqing, China. Our findings reveal a notably low vaccination intention rate (36.02%). Structural equation modeling identified severity perception, response cost, and self-efficacy as the three PMT constructs that directly influenced vaccination intention. Furthermore, knowledge and demographic factors demonstrated both direct and indirect effects, with their influence primarily mediated through these core PMT constructs.

The observed vaccination intention rate of 36.02% is substantially lower than the global average of 56–61% reported in recent meta-analyses (11, 30). This places our study population at the lower end of the spectrum of reported rates (8.8%–93.6%) (12, 31). Several factors may explain this discrepancy. First, our study sampled the general population, whereas higher rates are typically observed in high-risk groups such as the LGBTI community (84%) (11). Second, contextual differences in epidemic severity, public health strategies, and cultural factors significantly influence vaccination decisions (25). During the survey period (March–April 2025), no monkeypox vaccine had been approved for marketing in China, and vaccination was not routinely recommended for the general population; vaccination strategies were primarily targeted at high-risk groups. It is worth noting that on April 8, 2025, China’s first monkeypox vaccine (an MVA-strain live-attenuated monkeypox vaccine independently developed by the Shanghai Institute of Biological Products of China National Biotec Group) officially began recruiting participants for its Phase I clinical trial, with plans to enroll 120 volunteers aged 18 and older. This marked a significant step toward vaccine accessibility, but the vaccine was not yet available to the general public. Therefore, the low willingness rate observed among the general population in this study may partly reflect perceived low personal risk and the lack of public health vaccination recommendations, rather than pure vaccine hesitancy. Trust in the health sector and the perception of vaccination as a social responsibility have been identified as key drivers of vaccination intention (32).

The study found that severity perception of monkeypox was independently and positively associated with vaccination intention, which was consistent with previous studies (33–36). This finding aligns with the core conceptualization of protection motivation theory, which suggests that individuals are more likely to engage in health-promoting behaviors if they perceive a disease to be serious and to have serious consequences (20). During the monkeypox outbreak, individuals who perceived a potential risk of developing severe symptoms following infection were more inclined to receive vaccination to mitigate their risk (29, 34). Furthermore, studies have also indicated that severity perception not only influences individuals’ willingness to adopt personal protective measures but also promotes, to a certain extent, their acceptance of public health interventions, such as self-isolation and symptom monitoring (37).

Our analysis identified response cost as a major barrier to monkeypox vaccination. This finding is consistent with the broader literature on health behaviors, which conceptualizes response cost as encompassing both practical and psychological dimensions. Practical obstacles such as time commitment and financial concerns have been consistently linked to vaccine hesitancy (38), while psychological barriers rooted in concerns about safety, efficacy, and insufficient knowledge similarly undermine vaccination intention (32).

The present study demonstrated a significant association between self-efficacy and vaccination intention. This finding is consistent with other PMT research on different vaccines (21, 22, 39, 40). Likewise, a lack of self-efficacy was identified as a factor heightening vaccine hesitancy (25). Perceived self-efficacy denotes an individual’s confidence in their ability to organize and execute the courses of action required to manage challenging situations (41). For single-act health behaviors like vaccination, self-efficacy serves as a critical predictor of both behavioral intention and subsequent action (42).

Consistent with PMT, knowledge did not directly affect intention but exerted its influence indirectly by enhancing both threat appraisal (e.g., risk perception) and coping appraisal (e.g., self-efficacy) (43, 44). Misinformation may be a barrier to increasing vaccine uptake (45). Detailed and specific information from medical professionals and authorities can reduce misunderstandings and increase public confidence in vaccination by emphasizing the effectiveness and safety of vaccines, as compared to information from the Internet (46).

In addition, we found that age was negatively associated with vaccination intention. Similarly, a previous study also found that younger individuals exhibited a greater openness to vaccination (14). One possible explanation is that younger individuals hold greater confidence in scientific and technological advancements, in contrast to their older counterparts (47). Additionally, the higher cost of obtaining information for the middle-aged and elderly population makes them passive recipients of healthcare services, and the resulting lack of information can significantly affect an individual’s level of trust and willingness to be vaccinated (48).

## 5 Limitations

This study has the following limitations. First, the cross-sectional study design does not allow for inferring a causal relationship between the PMT constructs and vaccination intent. Second, although three administrative districts were selected at random, participant recruitment used convenience sampling via an online platform, which may have introduced selection bias (e.g., over-representation of young, highly educated, and digitally active individuals); consequently, the generalizability of the study results to older adults, rural populations, and those with limited internet access is limited. Third, all data were self-reported, which may be subject to reporting inaccuracies. Fourth, this study measured vaccination intent rather than actual vaccination behavior; although intent is a strong predictor of behavior, the gap between intent and actual behavior should be considered when interpreting the results. Fifth, during the survey period (March–April 2025), no monkeypox vaccine had been approved for marketing in China, nor was vaccination routinely recommended for the general population; the low intention rate may partly reflect this policy context and vaccine accessibility rather than pure vaccine hesitancy. Sixth, in the measurement model of this study, the validity of the construct “response efficacy” did not meet ideal standards; future research should consider using more well-established items to measure this construct. Finally, because online survey platforms cannot track nonrespondents, this study did not assess nonresponse bias. Future research should employ probability sampling, a longitudinal design, and more refined measurement tools to validate and expand upon the findings of this study.

## 6 Conclusion

This study provides empirical support for the Protection Motivation Theory in explaining monkeypox vaccination intention among Chinese adults. Our findings demonstrate that vaccination intention is shaped by a cognitive appraisal process wherein response cost constitutes the primary barrier, while severity perception and self-efficacy serve as positive determinants. Knowledge influences intention indirectly through these psychological mechanisms. These findings suggest that if monkeypox vaccines are included in routine recommendations for the general population in the future, developing comprehensive strategies to address both perceived threats and practical barriers will be crucial for increasing vaccination rates. Future research should further explore how willingness to be vaccinated translates into actual vaccination behavior across different populations and under different policy contexts.

## Data Availability

The raw data supporting the conclusions of this article will be made available by the authors, without undue reservation.

## References

[1] Cheng K Zhou Y Wu H . Bibliometric analysis of global research trends on monkeypox: Are we ready to face this challenge? J Med Virol. (2023) 95:e27892. doi: 10.1002/jmv.27892 35644836

[2] Magnus PV Andersen EK Petersen KB Birch-Andersen A . A POX-LIKE disease in cynomolgus monkeys. Acta Pathologica Microbiologica Scandinavica. (1959) 46:156–76. doi: 10.1111/j.1699-0463.1959.tb00328.x 40046247

[3] Ladnyj ID Ziegler P Kima E . A human infection caused by monkeypox virus in Basankusu Territory, Democratic Republic of the Congo. Bull World Health Organ. (1972) 46:593–7. PMC2480792 4340218

[4] Kumar N Ahmed F Raza MS Rajpoot PL Rehman W Khatri SA . Monkeypox cross-sectional survey of knowledge, attitudes, practices, and willingness to vaccinate among university students in Pakistan. Vaccines (Basel). (2022) 11:97. doi: 10.3390/vaccines11010097 36679942 PMC9862138

[5] Cabanillas B Murdaca G Guemari A Torres MJ Azkur AK Aksoy E . Monkeypox 2024 outbreak: Fifty essential questions and answers. Allergy. (2024) 79:3285–309. doi: 10.1111/all.16374 39495103

[6] WHO Director-General declares mpox outbreak a public health emergency of international concern. Saudi Med J. (2024) 45:1002–3. doi: 10.15537/1658-3175.8449 PMC11376700 39218470

[7] Zhao H Wang W Zhao L Ye S Song J Lu R . The first imported case of monkeypox in the mainland of China - Chongqing Municipality, China, September 16, 2022. China CDC Wkly. (2022) 4:853–4. doi: 10.46234/ccdcw2022.175 36284686 PMC9579970

[8] Ren R Li C Bai W Wang Y Li D Ding F . The epidemiological characteristics of mpox cases - China, 2023. China CDC Wkly. (2024) 6:619–23. doi: 10.46234/ccdcw2024.118 38966310 PMC11219300

[9] Wong LP Alias H Lee HY Zhao Q Huang Z Hu Z . Investigating post-COVID-19 confidence in emergency use authorization vaccines: A hypothetical case of mpox. PLoS Negl Trop Dis. (2025) 19:e0013037. doi: 10.1371/journal.pntd.0013037 40440224 PMC12187215

[10] Liu H Wang W Zhang Y Wang F Duan J Huang T . Global perspectives on smallpox vaccine against monkeypox: A comprehensive meta-analysis and systematic review of effectiveness, protection, safety and cross-immunogenicity. Emerg Microbes Infect. (2024) 13:2387442. doi: 10.1080/22221751.2024.2387442 39082272 PMC11332295

[11] Ulloque-Badaracco JR Alarcón-Braga EA Hernandez-Bustamante EA Al-kassab-Córdova A Benites-Zapata VA Bonilla-Aldana DK . Acceptance towards monkeypox vaccination: A systematic review and meta-analysis. Pathogens. (2022) 11:1248. doi: 10.3390/pathogens11111248 36364999 PMC9697127

[12] Riad A Drobov A Rozmarinová J Drapáčová P Klugarová J Dušek L . Monkeypox knowledge and vaccine hesitancy of Czech healthcare workers: A health belief model (HBM)-based study. Vaccines (Basel). (2022) 10:2022. doi: 10.3390/vaccines10122022 36560432 PMC9788212

[13] Peptan C Băleanu VD Mărcău FC . Study on the vaccination of the population of Romania against monkeypox in terms of medical security. Vaccines (Basel). (2022) 10:1834. doi: 10.3390/vaccines10111834 36366343 PMC9697308

[14] Wang B Peng X Li Y Fu L Tian T Liang B . Perceptions, precautions, and vaccine acceptance related to monkeypox in the public in China: A cross-sectional survey. J Infect Public Health. (2023) 16:163–70. doi: 10.1016/j.jiph.2022.12.010 36535136 PMC9743792

[15] Dong C Yu Z Zhao Y Ma X . Knowledge and vaccination intention of monkeypox in China's general population: A cross-sectional online survey. Travel Med Infect Dis. (2023) 52:102533. doi: 10.1016/j.tmaid.2022.102533 36543284 PMC9759477

[16] Liu S Chu H . Parents' COVID-19, HPV, and monkeypox vaccination intention: A multilevel structural equation model of risk, benefit, barrier, and efficacy perceptions and individual characteristics. Patient Educ Couns. (2023) 114:107842. doi: 10.1016/j.pec.2023.107842 37301013 PMC10246936

[17] daCosta DiBonaventura M Chapman GB . Moderators of the intention–behavior relationship in influenza vaccinations: Intention stability and unforeseen barriers. Psychol Health. (2005) 20:761–74. doi: 10.1080/14768320500183368 37339054

[18] Griffin B Conner M Norman P . Applying an extended protection motivation theory to predict Covid-19 vaccination intentions and uptake in 50-64 year olds in the UK. Soc Sci Med. (2022) 298:114819. doi: 10.1016/j.socscimed.2022.114819 35245755 PMC8867961

[19] Alshahrani NZ Alshahrani SM Farag S Rashid H . Domestic Saudi Arabian travellers' understanding about COVID-19 and its vaccination. Vaccines (Basel). (2021) 9:895. doi: 10.3390/vaccines9080895 34452020 PMC8402648

[20] Rogers RW . A protection motivation theory of fear appeals and attitude change. J Psychol. (1975) 91:93–114. doi: 10.1080/00223980.1975.9915803 28136248

[21] Ling M Kothe EJ Mullan BA . Predicting intention to receive a seasonal influenza vaccination using protection motivation theory. Soc Sci Med. (2019) 233:87–92. doi: 10.1016/j.socscimed.2019.06.002 31195194

[22] Eberhardt J Ling J . Explaining COVID-19 vaccination intention in younger adults using protection motivation theory. Health Psychol. (2023) 42:577–83. doi: 10.1037/hea0001231 35980723

[23] Liu C Nicholas S Wang J . The association between protection motivation and hepatitis b vaccination intention among migrant workers in Tianjin, China: A cross-sectional study. BMC Public Health. (2020) 20:1219. doi: 10.1186/s12889-020-09292-2 32778075 PMC7418384

[24] Nie R Ma Z Rui X Li T . HPV vaccination willingness and behavior among nursing female students in China based on the protection motivation theory: A cross-sectional study. Hum Vaccin Immunother. (2025) 21:2569738. doi: 10.1080/21645515.2025.2569738 41056529 PMC12505507

[25] Gao Y Liu S Xu H Wang Y Xu G Hu F . The mpox vaccine hesitancy scale for mpox: Links with vaccination intention among men who have sex with men in six cities of China. Vaccines (Basel). (2024) 12:1009. doi: 10.3390/vaccines12091009 39340039 PMC11436122

[26] Di Giulio DB Eckburg PB . Human monkeypox: An emerging zoonosis. Lancet Infect Dis. (2004) 4:15–25. doi: 10.1016/s1473-3099(03)00856-9 14720564 PMC9628772

[27] Huang PC Hung CH Kuo YJ Chen YP Ahorsu DK Yen CF . Expanding protection motivation theory to explain willingness of COVID-19 vaccination uptake among Taiwanese university students. Vaccines (Basel). (2021) 9:1046. doi: 10.3390/vaccines9091046 34579283 PMC8473221

[28] Wang J Li T Ge J Zhou M Walker AN Chen J . Applying two behavioral theories to predict the willingness to receive COVID-19 vaccine booster in the elderly: A cross-sectional study. Res Soc Adm Pharm. (2023) 19:495–501. doi: 10.1016/j.sapharm.2022.10.011 36357271 PMC9632265

[29] Walsh-Buhi ML Houghton RF Valdez D Walsh-Buhi ER . A theory-based assessment of mpox: Findings from a nationally representative survey of U.S. adults. PLoS One. (2024) 19:e0299599. doi: 10.1371/journal.pone.0299599 38489274 PMC10942057

[30] León-Figueroa DA Barboza JJ Valladares-Garrido MJ Sah R Rodriguez-Morales AJ . Prevalence of intentions to receive monkeypox vaccine. A systematic review and meta-analysis. BMC Public Health. (2024) 24:35. doi: 10.1186/s12889-023-17473-y 38166776 PMC10763398

[31] Harapan H Setiawan AM Yufika A Anwar S Wahyuni S Asrizal FW . Physicians' willingness to be vaccinated with a smallpox vaccine to prevent monkeypox viral infection: A cross-sectional study in Indonesia. Clin Epidemiol Glob Health. (2020) 8:1259–63. doi: 10.1016/j.cegh.2020.04.024 36245638 PMC9533989

[32] Alarifi AM Alshahrani NZ Sah R . Are Saudi healthcare workers willing to receive the monkeypox virus vaccine? Evidence from a descriptive-baseline survey. Trop Med Infect Dis. (2023) 8:396. doi: 10.3390/tropicalmed8080396 37624334 PMC10459197

[33] Luo S Jiao K Zhang Y Xu Y Zhou J Huang S . Behavioral intention of receiving monkeypox vaccination and undergoing monkeypox testing and the associated factors among young men who have sex with men in China: Large cross-sectional study. JMIR Public Health Surveill. (2024) 10:e47165. doi: 10.2196/47165 38502181 PMC10988377

[34] Dukers-Muijrers N Evers Y Widdershoven V Davidovich U Adam PCG Op de Coul ELM . Mpox vaccination willingness, determinants, and communication needs in gay, bisexual, and other men who have sex with men, in the context of limited vaccine availability in the Netherlands (Dutch Mpox-survey). Front Public Health. (2023) 10:1058807. doi: 10.3389/fpubh.2022.1058807 36684959 PMC9850232

[35] Evers YJ Schneider F Widdershoven V Goense CJD Peters CMM van Elsen SG . Using a theoretical framework of intervention mapping to inform public health communication messages designed to increase vaccination uptake; the example of mpox in the Netherlands. BMC Public Health. (2023) 23:2373. doi: 10.1186/s12889-023-17311-1 38037024 PMC10688141

[36] Reyes-Urueña J D'Ambrosio A Croci R Bluemel B Cenciarelli O Pharris A . High monkeypox vaccine acceptance among male users of smartphone-based online gay-dating apps in Europe, 30 July to 12 August 2022. Euro Surveill. (2022) 27:2200757. doi: 10.2807/1560-7917.es.2022.27.42.2200757 36268737 PMC9585877

[37] May T Towler L Smith LE Horwood J Denford S Rubin GJ . Mpox knowledge, behaviours and barriers to public health measures among gay, bisexual and other men who have sex with men in the UK: A qualitative study to inform public health guidance and messaging. BMC Public Health. (2023) 23:2265. doi: 10.1186/s12889-023-17196-0 37978506 PMC10655366

[38] Faryabi R Rezabeigi Davarani F Daneshi S Moran DP . Investigating the effectiveness of protection motivation theory in predicting behaviors relating to natural disasters, in the households of southern Iran. Front Public Health. (2023) 11:1201195. doi: 10.3389/fpubh.2023.1201195 37744489 PMC10513462

[39] Gao Y Wei T Gong R Cai L Sheng Y Shang M . Development of a herpes zoster vaccination intention scale and identification of factors associated with vaccine hesitancy among middle-aged and older attendees in community health centers: A protection motivation theory based study. Hum Vaccin Immunother. (2025) 21:2516947. doi: 10.1080/21645515.2025.2516947 40524390 PMC12184168

[40] Wu F Yuan Y Deng Z Yin D Shen Q Zeng J . Acceptance of COVID-19 booster vaccination based on the protection motivation theory: A cross-sectional study in China. J Med Virol. (2022) 94:4115–24. doi: 10.1002/jmv.27825 35506329 PMC9348068

[41] Ansari-Moghaddam A Seraji M Sharafi Z Mohammadi M Okati-Aliabad H . The protection motivation theory for predict intention of COVID-19 vaccination in Iran: A structural equation modeling approach. BMC Public Health. (2021) 21:1165. doi: 10.1186/s12889-021-11134-8 34140015 PMC8209774

[42] Fall E Izaute M Chakroun-Baggioni N . How can the health belief model and self-determination theory predict both influenza vaccination and vaccination intention? A longitudinal study among university students. Psychol Health. (2018) 33:746–64. doi: 10.1080/08870446.2017.1401623 29132225

[43] Huang R Wang Z Yuan T Nadarzynski T Qian HZ Li P . Using protection motivation theory to explain the intention to initiate human papillomavirus vaccination among men who have sex with men in China. Tumour Virus Res. (2021) 12:200222. doi: 10.1016/j.tvr.2021.200222 34175495 PMC8261654

[44] Bai Y Liu Q Chen X Gao Y Gong H Tan X . Protection motivation theory in predicting intention to receive cervical cancer screening in rural Chinese women. Psychooncology. (2018) 27:442–9. doi: 10.1002/pon.4510 28749603

[45] Husted M Gibbons A Cheung WY Keating S . COVID-19 vaccination hesitancy in adults in the United Kingdom: Barriers and facilitators to uptake. Health Psychol. (2023) 42:584–92. doi: 10.1037/hea0001256 36633991

[46] Fan J Wang Q Deng Y Liang J Walker AN You H . Explanation of intention toward influenza vaccination among cardiovascular disease patients: An application of the extended protection motivation theory. Public Health. (2025) 242:228–35. doi: 10.1016/j.puhe.2025.03.010 40132460

[47] Magadmi RM Kamel FO . Beliefs and barriers associated with COVID-19 vaccination among the general population in Saudi Arabia. BMC Public Health. (2021) 21:1438. doi: 10.1186/s12889-021-11501-5 34289817 PMC8294288

[48] Wang X Xing Y Zhang E Dai Z Li Y Shang S . Understanding herpes zoster vaccine hesitancy and information asymmetry: A qualitative study in China. Front Public Health. (2024) 12:1429522. doi: 10.3389/fpubh.2024.1429522 39286749 PMC11402811

