# Supplementary material for: Using protection motivation theory to predict monkeypox vaccination intention among adults in Chongqing, China
Source: Front Immunol. 2026 Aug 17;17:1871884. doi: 10.3389/fimmu.2026.1871884 (PMC13527038; doi:10.3389/fimmu.2026.1871884)
Supplement: Supplementary file 1 [file Table1.docx]

Supplementary Table S1: CR and AVE for Each Construct

| Construct | CR | AVE |
| --- | --- | --- |
| Risk Perception | 0.857 | 0.674 |
| Severity Perception | 0.890 | 0.669 |
| Fear Arousal | 0.851 | 0.590 |
| Response Efficacy | 0.663 | 0.331 |
| Response Cost | 0.822 | 0.541 |
| Self-Efficacy | 0.927 | 0.719 |

Supplementary Table S2. Full list of PMT items

| Construct | Item |
| --- | --- |
| Risk perception | RP1 I think I’m at risk of contracting monkeypox |
|  | RP2 I think my family is at risk of contracting monkeypox. |
|  | RP3 Even if you’re in good health, you can still contract monkeypox |
| Severity perception | SP1 I think monkeypox is a serious health issue. |
|  | SP2 Contracting monkeypox can lead to serious long-term complications and may even be life-threatening. |
|  | SP3 Contracting monkeypox can interfere with work or school. |
|  | SP4 Contracting monkeypox would have a huge impact on my entire family. |
| Fear arousal | FA1 I’m afraid to talk to others about monkeypox. |
|  | FA2 Just thinking about monkeypox makes me feel uneasy. |
|  | FA3 I can’t sleep because I’m worried about contracting monkeypox. |
|  | FA4 I’m afraid of dying from monkeypox. |
| Response efficacy | RE1 Getting vaccinated against monkeypox can alleviate my current anxiety about the monkeypox outbreak. |
|  | RE2 Getting vaccinated against monkeypox can provide additional health benefits. |
|  | RE3 Getting vaccinated against monkeypox can reduce the risk of contracting monkeypox. |
|  | RE4 Getting vaccinated against monkeypox can help me focus better on work and studies. |
| Response cost | RC1 Getting the monkeypox vaccine may cause side effects. |
|  | RC2 The process of getting the monkeypox vaccine may increase my risk of contracting monkeypox. |
|  | RC3 Getting the monkeypox vaccine may not be effective in preventing infection. |
|  | RC4 Getting the monkeypox vaccine is time-consuming. |
| Self-efficacy | SE1 I would get the monkeypox vaccine even if no one around me had monkeypox. |
|  | SE2 I would get the monkeypox vaccine even if I were very busy at work. |
|  | SE3 I would get the monkeypox vaccine even if the vaccination site were far away. |
|  | SE4 I would get the monkeypox vaccine even if I had to pay for it myself. |
|  | SE5 I would get the monkeypox vaccine even if my family and friends disapproved. |

Supplementary Table S3. Discriminant validity of the measurement model (Fornell-Larcker criterion)

| Construct | RP | SP | FA | RE | RC | SE |
| --- | --- | --- | --- | --- | --- | --- |
| Risk perception (RP) | **0.82** |  |  |  |  |  |
| Severity perception (SP) | 0.33 | **0.82** |  |  |  |  |
| Fear arousal (FA) | 0.24 | 0.33 | **0.77** |  |  |  |
| Response efficacy (RE) | 0.38 | 0.60 | 0.44 | **0.58** |  |  |
| Response cost (RC) | 0.20 | 0.27 | 0.55 | 0.34 | **0.74** |  |
| Self-efficacy (SE) | 0.40 | 0.39 | 0.32 | 0.63 | 0.24 | **0.85** |

Note: Diagonal elements (bold) are the square roots of the average variance extracted (AVE). Off-diagonal elements are the Pearson correlation coefficients between constructs (from Table 4). Discriminant validity is supported when the diagonal value exceeds the off-diagonal values in the corresponding row and column. RE did not meet the Fornell-Larcker criterion, as its √AVE (0.58) was smaller than its correlation coefficient with SE (r = 0.63, shown in the table above), indicating limited discriminant validity between RE and SE.

Supplementary Table S4. Standardized and unstandardized path coefficients of the structural equation model

| Path | Unstandardized β | S.E. | C.R. | P | Standardized β |
| --- | --- | --- | --- | --- | --- |
| **PMT constructs → Vaccination intention** |  |  |  |  |  |
| Risk perception → Intention | 1.00 | - | - | - | 0.86 |
| Severity perception → Intention | 0.22 | 0.07 | 3.05 | 0.002 | 0.14 |
| Fear arousal → Intention | 0.10 | 0.06 | 1.58 | 0.115 | 0.07 |
| Response efficacy → Intention | 0.09 | 0.12 | 0.76 | 0.446 | 0.05 |
| Response cost → Intention | -0.60 | 0.10 | -5.97 | <0.001 | -0.26 |
| Self-efficacy → Intention | 0.16 | 0.07 | 2.30 | 0.021 | 0.13 |
| **Demographics → Vaccination intention** |  |  |  |  |  |
| Age → Intention | -0.01 | 0.00 | -3.14 | 0.002 | -0.10 |
| **Demographics → PMT constructs** |  |  |  |  |  |
| Knowledge → Risk perception | 0.02 | 0.01 | 1.97 | 0.048 | 0.06 |
| Knowledge → Severity perception | 0.06 | 0.01 | 7.77 | <0.001 | 0.28 |
| Knowledge → Response efficacy | 0.04 | 0.01 | 5.46 | <0.001 | 0.16 |
| Knowledge → Response cost | -0.02 | 0.01 | -3.09 | 0.002 | -0.11 |
| Knowledge → Self-efficacy | 0.05 | 0.01 | 4.95 | <0.001 | 0.18 |
| Age → Severity perception | 0.00 | 0.00 | -2.60 | 0.009 | -0.09 |
| Age → Fear arousal | 0.00 | 0.00 | -0.83 | 0.006 | 0.12 |
| Income → Severity perception | 0.04 | 0.01 | 3.31 | <0.001 | 0.09 |
| Income → Response efficacy | 0.03 | 0.01 | 2.68 | 0.007 | 0.08 |
| Income → Self-efficacy | 0.04 | 0.02 | 2.68 | 0.007 | 0.09 |

Note: S.E. = standard error; C.R. = critical ratio (estimate/S.E.).

Supplementary Table S5. Bootstrap indirect effects and 95% confidence intervals

| **Path** | **Indirect Effect** | **Bootstrap 95% CI** | **p-value** |
| --- | --- | --- | --- |
| **Knowledge → PMT constructs → Intention** |  |  |  |
| **Knowledge** → RP → Intention | **0.009** | **[0.004, 0.015]** | **0.001** |
| **Knowledge** → SP → Intention | 0.006 | [-0.001, 0.014] | 0.109 |
| **Knowledge** → FA → Intention | 0.000 | [-0.001, 0.003] | 0.755 |
| **Knowledge** → RE → Intention | **0.013** | **[0.005, 0.022]** | **0.002** |
| Knowledge → RC → Intention | 0.004 | [0.000, 0.010] | 0.079 |
| **Knowledge** → SE → Intention | **0.020** | **[0.012, 0.029]** | **<0.001** |
| **Income → PMT constructs → Intention** |  |  |  |
| Income → RP→ Intention | 0.005 | [-0.001, 0.013] | 0.182 |
| Income → SP → Intention | 0.004 | [-0.000, 0.012] | 0.172 |
| Income → FA → Intention | 0.000 | [-0.003, 0.002] | 0.998 |
| Income → RE → Intention | **0.009** | **[0.002, 0.020]** | **0.043** |
| Income → RC → Intention | -0.003 | [-0.012, 0.003] | 0.407 |
| Income → SE → Intention | **0.017** | **[0.005, 0.031]** | **0.011** |
| **Age → PMT constructs → Intention** |  |  |  |
| Age → RP→ Intention | 0.000 | [-0.001, 0.001] | 0.667 |
| Age → SP → Intention | -0.000 | [-0.001, 0.000] | 0.195 |
| Age → FA → Intention | 0.000 | [-0.000, 0.001] | 0.625 |
| Age → RE → Intention | 0.000 | [-0.001, 0.001] | 0.826 |
| Age → RC → Intention | 0.001 | [-0.000, 0.002] | 0.249 |
| Age → SE → Intention | -0.000 | [-0.002, 0.001] | 0.756 |

Note: Bootstrap 95% confidence intervals were estimated using 5,000 bootstrap resamples with bias-corrected percentile method. Bold font indicates statistically significant indirect effects. RP = risk perception; SP = severity perception; FA = fear arousal; RE = response efficacy; RC = response cost; SE = self-efficacy.
